# Nonreciprocal nonlinear wave scattering by loss-compensated active hyperbolic structures

**DOI:** 10.1038/srep42919

**Published:** 2017-02-22

**Authors:** O. V. Shramkova, G. P. Tsironis

**Affiliations:** 1 Crete Center for Quantum Complexity and Nanotechnology, Department of Physics, University of Crete P.O. Box 2208, 71003 Heraklion, Greece; 2 Institute of Electronic Structure and Laser, Foundation for Research and Technology–Hellas, P.O. Box 1527, 71110 Heraklion, Greece; 3 National University of Science and Technology MISiS, Leninsky prosp. 4, Moscow, 119049, Russia

## Abstract

The combinatorial frequency generation (CFG) in active periodic semiconductor-dielectric structures has been explored through illumination by a pair of pump waves with dissimilar frequencies and incidence angles. We study the influence of gain on linear refraction properties of the stack and on the efficiency of the mixing processes by the system with the resistive character of nonlinearity. We demonstrate that the introduction of gain dielectric material inside the stack not only compensates for losses caused by the collisions of the electrons in semiconductor media but also improves the efficiency of the CFG. We show that in systems with weak asymmetry of linear response we can get significant nonreciprocity of nonlinear interaction.

Recent theoretical and experimental studies of nonlinear metamaterials and photonic crystals have shown that these artificial materials with nonlinear inclusions can be instrumental for increasing the efficiency of frequency conversion and harmonic generation in THz and optical ranges^1^,^2^,^3^. The distinctive features of nonlinear artificial media are associated with the possibility of dispersion engineering by means of changing the microscopic properties and geometry of its constituent particles. However, the dissipation in a medium limits the overall functionality of the system^4^. It has been already demonstrated that the second harmonic generation enhancement at the band edges is noticeably impaired by the higher losses of the pump wave that reduce the overall conversion efficiency^5^. In refs 6,7 it was shown that the combined effect of the pump wave dissipation and attenuation of the mixing products passing through the periodic and quasi-periodic stacks of binary layers of anisotropic nonlinear dielectrics causes dramatic reduction of the CFG efficiency. One way to mitigate this problem is to use the active materials, so that to compensate the losses with the gain of active media. Loss compensation in linear active metal-dielectric photonic band gap structures was considered in refs 8, 9, 10, 11, 12. In fact, loss compensation in such materials opens new routes towards the design of gain-assisted low-loss hyperbolic materials. For example, the introduction of the gain can enhance the lens peculiarities also for wavelengths where the lens performance is frustrated^9^. The nonlinearity of semiconductor layers is directly related to the losses inflicted by collisions of carriers^13^. Therefore in contrast to the dielectrics where losses reduce the efficiency of nonlinear mixing, the intensities of the waves with combinatorial frequency emitted from the same stack grow with the collision frequency in semiconductor layers. Thus, the issue is to see whether or not the introduction of gain within the dielectric layers affects the efficiency of wave mixing by the semiconductor-dielectric stack.

One of the most relevant attributes of the fine-stratified periodic semiconductor-dielectric media is the hyperbolic-shaped dispersion, which can cause negative refraction. The hyperbolic materials have since become a very active research area, enabling numerous applications including subwavelength imaging, nanolithography and emission engineering. For such materials the isofrequency contours of the plane waves have a hyperbolic shape which allows the propagation of waves with infinitely large wave numbers^14^,^15^. The properties of hyperbolic materials can be described by the effective parameters derived in the frameworks of the effective medium approximation under the assumption that constitutive elements of the composite are much smaller then the wavelength. Recent studies have indicated that hyperbolic materials have significant potential for enhancement of the effects associated with the second- and third-order nonlinearities^12^,^16^,^17^. The quasi-phase-matching condition is the necessary prerequisite of nonlinear mixing that essentially enforces conservation of linear momentum in the wave interactions. It was demonstrated that the dispersion afforded by hyperbolic metamaterials facilitates the phase synchronism between the interacting waves.

The aim of this paper is to explore the possibility of the nonlinear activity enhancement in the loss-compensated semiconductor-dielectric multilayers. Here the properties of the combinatorial frequencies generated by the active nonlinear semiconductor-dielectric photonic crystal illuminated by the plane waves of two tones are investigated. It was shown that for such non-Hermitian structure with active layers both the linear and nonlinear responses become dependent on the direction of pump wave incidence.

## Results

### Configuration description

**Figure 1:**
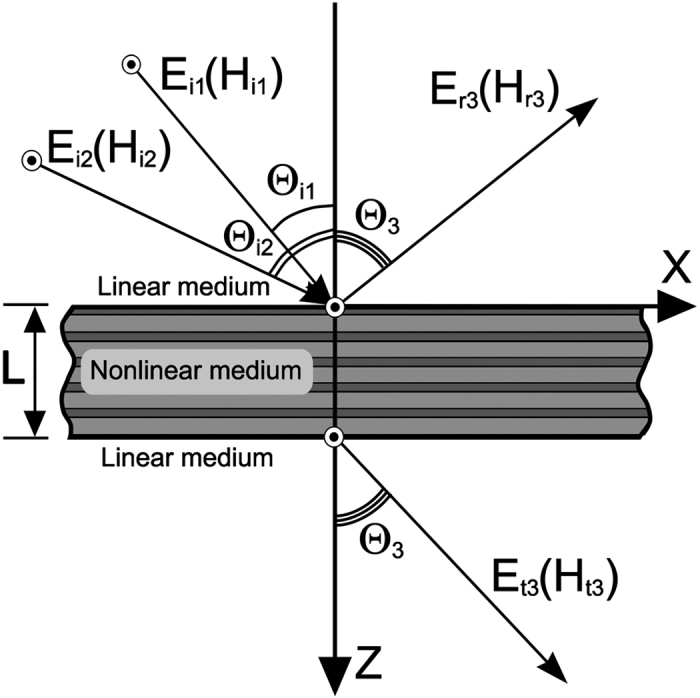
Geometry of the problem.

The periodic structure shown in Fig. 1 is composed of stacked semiconductor and dielectric layers with thicknesses *d*_1_ and *d*_2_, respectively. The unit cell size of periodic stack (*d* = *d*_1_ + *d*_2_) is much smaller than the free-space wavelength. The stack of the total thickness *L* = *N·d* contains *N* unit cells and is surrounded by linear homogeneous medium with dielectric permittivity *ε*_*a*_ at *z* ≤ 0 and *z* ≥ *L*. The system is assumed of infinite extent in the *x0y*-plane.

The stack is illuminated by a pair of pump plane waves of frequencies *ω*_1_ and *ω*_2_ incident at angles Θ_*i*1_ and Θ_*i*2_, respectively. Since the layers are assumed isotropic in the *xOy* plane, incident waves of the TE and TM polarisations with the fields independent of the *y*-coordinate can be analysed separately. The constituent semiconductor layers are represented as solid-state plasma with mobile charges. Then in a self-consistent formulation of the nonlinear scattering problem, Maxwell’s equations are solved simultaneously with the hydrodynamic and current continuity equations describing the charge dynamics.


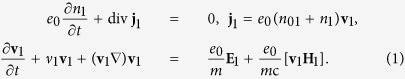


where *c* is the speed of light in free space, *e*_0_ and *m* are the carrier charge and mass; *n*_01_ is the carrier concentration at equilibrium; **v**_1_, **j**_1_ and *n*_1_ are the velocity, current density and variable carrier concentration in each semiconductor layer, respectively. Inspection of these equations in the case of the TE polarised waves shows that the semiconductor layers remain linear. As the result, we consider only the TM waves scattering by the stacks of semiconductor layers.

In the long-wavelength limit the dispersion relation for a linear stratified semiconductor-dielectric structure takes the form refs 18,19


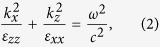


where *k*_*x*_ and *k*_*z*_ are the transverse and longitudinal wavenumbers of uniaxial medium with the effective components of dielectric permittivity tensor


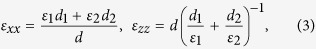


where 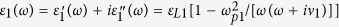 is the equivalent permittivity of semiconductor layer, 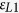 is the lattice permittivity; *ν*_1_ is the collision frequency; 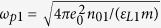 is the plasma frequency, 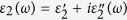 is the dielectric permittivity of dielectric layer. For the active dielectric medium, we take the gain values 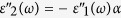, 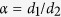. The analysis of the effective components *ε*_*xx*_ and *ε*_*zz*_ for this non-Hermitian system demonstrates that such form of dielectric permittivity for the active dielectric medium provides the lossless component *ε*_*xx*_. At the same time the component *ε*_*zz*_ has an imaginary part which exhibits noticeable influence on the longitudinal wavenumber of the uniaxial fine-stratified semiconductor medium only at *ω* ≈ *ω*_*p*1_. This influence is negligible at small angles of incidence. Below the plasma frequencies, the permittivity of semiconductor is negative. As the result, two hyperbolic regimes for which the effective dielectric permittivity components have opposite signs in two orthogonal directions can be attained at frequencies *ω* < *ω*_*p*1_. The frequency bounds of hyperbolic regimes, *ω*_*g*1_ (*ε*_*xx*_ = 0) and *ω*_*g*2_ (*ε*_*zz*_ → ∞), are defined as follows


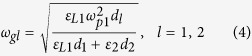


For *α* < 1 the first hyperbolic regime with *ε*_*xx*_ < 0, *ε*_*zz*_     > 0 corresponds to *ω* *<* *ω*_*g*1_, the second regime (*ε*_*xx*_ > 0, *ε*_*zz*_ < 0) is observed at *ω*_*g*2_ < *ω* < *ω*_p1_. For the frequency band *ω*_*g*1_ < *ω* < *ω*_*g*2_ both components are positive and the contour of constant frequency is an ellipse. If *α* > 1, the upper bound for first regime and lower bound for second one are exchanged. Moreover, at frequencies *ω*_*g*2_ < *ω* < *ω*_*g*1_ both components of the dielectric permittivity tensor are negative and the stack is completely opaque for incident waves. If the thicknesses of semiconductor layers are equal (*α* = 1), *ω*_*g*1_ = *ω*_*g*2_ and the gap between two different frequency bands for hyperbolic media will not be observed.

### Linear reflectance

**Figure 2:**
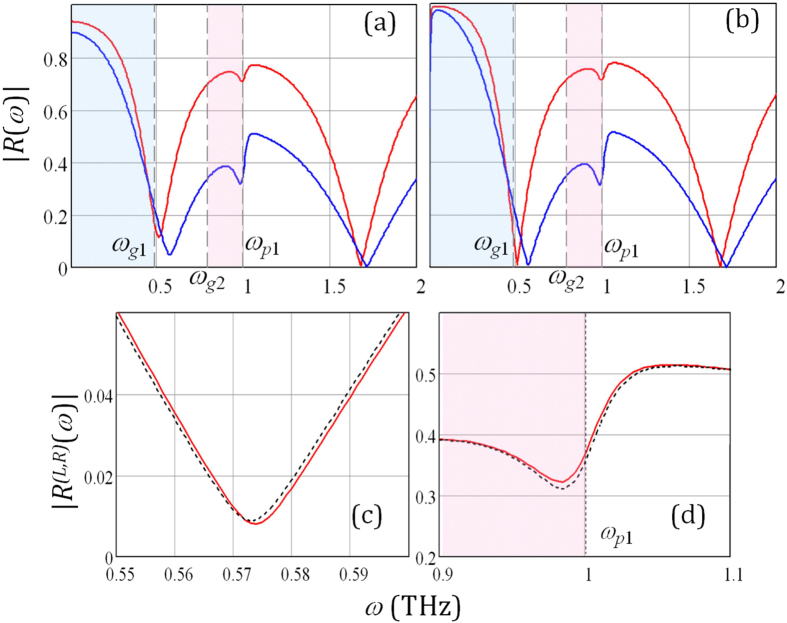
TM wave reflectance from the (**a**) - passive and (**b**–**d**) - active stacks with the default parameters, illuminated at the incidence angle Θ_i_ = 30° (solid red line) and Θ_i_ = 60° (solid blue line); (**c**,**d**) - Θ_i_ = 30°, |*R*^(*L*)^| -solid red curve, |*R*^(*R*)^| - dashed black curve.

The frequency mixing efficiency in the stacks of nonlinear layers essentially depends on the phase coherence of both pump waves and the nonlinear products, which is controlled by the linear dispersion and anisotropy of the constituent layers. To gain insight in the properties of CFG, it is necessary first to examine the stack linear reflectance *R(ω*) which is responsible for the pump wave amplitudes inside the layers. To analyse the scattering problem we use the Fresnel approach^20^ and the exact transmission matrix technique. The reflection coefficients (for left incidence – *R*^(*L*)^, for right incidence – *R*^(*R*)^) are readily obtained by satisfying the continuity conditions for the tangential field components at the stack interfaces. The reflectance of TM waves incident at slant angles Θ_*i*_ = 30° and Θ_*i*_ = 60° on the fine-stratified periodic stack with *N* = 40, d = 4 *μ*m*, α* = 3/7 is displayed in Fig. 2. The calculation was carried out for the lattice consisting of a GaAs semiconductor as the first layer inside the unit cell (*ε*_01_ = 10.9, *ω*_*p*1_ = 1 THz, *ν*_1_ = 0.05 THz) and dielectric material with 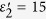 as the second slab. Such semiconductor-based structure is optimal for THz frequencies. The glass materials doped with laser-active ions can be used as gain dielectrics in experimental works. It was assumed that the stack is surrounded by air with the permittivity *ε*_*a*_ = 1.

The bands corresponding to different hyperbolic regimes are blue and pink shaded in Fig. 2. The comparison of the dependences for passive (Fig. 2a) and active (Fig. 2b) stacks demonstrates that the presence of the layer with gain can noticeably (more than 10%) reduce the dips of reflectivity at the frequency band corresponding to the elliptical regime (*ω*_*g*1_ < *ω* < *ω*_*g*2_). Specifically, for the loss-compensated stacks the increase of collision frequency *ν*_1_ leads to the movement of reflectivity dips towards lower frequencies corresponding to the first hyperbolic regime. The scattering properties of the stack are simulated at wavelengths that are about 10 times larger than the total thickness of the structure. The number of the resonance dips for linear reflectance will rise with the number of periods *N* or thickness of the period *d* at frequencies *ω* *>* *ω*_*g*1_. It is evident that in the linear active loss-compensated semiconductor-dielectric multilayers the reflectivity depends on the direction of wave incidence. As the result, the slight mismatch of the curves for |*R*^(*L*)^| and |*R*^(*R*)^| in the frequency band *ω*_*g*1_ < *ω* < *ω*_*g*2_ (Fig. 2c) and close to the frequency *ω*_*p*1_ (Fig. 2d) can be observed. Note that the transmittivity of such active system will be the same for both directions of wave incidence. In the next section we will demonstrate that the frequency mixing efficiency and nonreciprocity are determined by the pump wave intensities inside the layers which, in turn, depend on the stack linear reflectance. Thus, the weakly assymetric linear scattering can lead to the noticeable nonreciprocity of CFG by active hyperbolic structure.

### Three-wave mixing in a periodic stack of the layers

Assuming that nonlinearity of the constituent layers is weak and the three-wave mixing process is dominant, the characteristics of the TM waves of frequency *ω*_3_ = *ω*_1_ + *ω*_2_ generated inside the stacks can be obtained by the harmonic balance method. Then the fields *H*_*y*1_ of frequency *ω*_3_ in each nonlinear semiconductor layer are described by non-homogeneous Helmholtz equation^13^


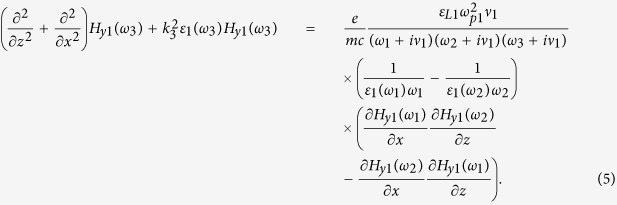


The analysis of Helmholtz equation (5) shows that away from plasma frequency, the frequency mixing process is primarily rendered by the resistive nonlinearity of semiconductor layer, directly proportional to collision frequency. At the same time, in the proximity of *ω*_*p*1_ the effect of resistive nonlinearity is minor. The solution of (5) composed of the particular and general solutions can be represented as a superposition of 6 waves, which vary with *z* as


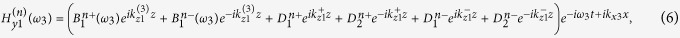


where superscript *n* is a serial number of a primitive cell, which identifies a semiconductor layer position in the stack, 
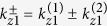
 and 
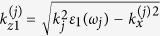
 (*j* = 1, 2, 3) are the transverse components of the refracted waves in the semiconductor layer. The longitudinal wave vector component 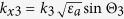 at the combinatorial frequency *ω*_3_ is determined by the requirement of the phase synchronism in the three-wave mixing process and 
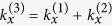
, where 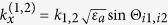, 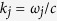. Angle Θ_3_ defines the direction of the combinatorial frequency emission from the stack as shown in Fig. 1.

The amplitude coefficients 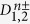 of the waves generated in semiconductor layers at frequency *ω*_3_ are expressed in terms of the field magnitudes in the layer at frequencies *ω*_1_ and *ω*_2_


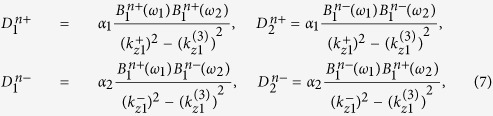


where


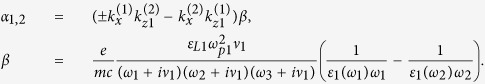


Here 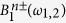 are the field amplitudes inside the semiconductor layer of the *n*^th^ period at the incident wave frequencies *ω*_1_ and *ω*_2_. Let us note that these amplitudes depend on the stack linear reflectance.

In order to examine the three-wave mixing process in the nonlinear layered structures, we generalise the transfer matrix method (TMM) based analysis^6^ for the case of two obliquely incident pump waves. The TMM can now be used for interrelating the fields of frequency *ω*_3_ at the layer interfaces. Enforcing the periodic boundary conditions through the whole periodic structure, the fields at the stack outer interfaces can be presented in the form


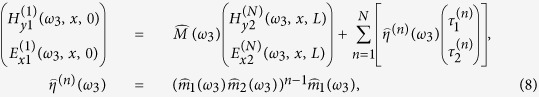


where 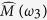 is the transfer matrix of the whole stack and 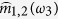 are the transfer matrices of the layers at combinatorial frequency *ω*_3_; 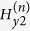 and 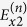 are the fields in linear dielectric layers (
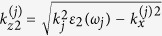
 are the *z* components of the wave vectors in dielectric layers at frequencies *ω*_*j*_); 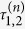 are expressed in terms of coefficients 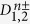 presented in (7):


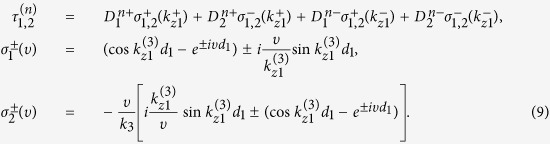


The reflected and refracted fields of the TM waves outgoing from the stack into the surrounding homogeneous media as the result of the nonlinear scattering at frequency *ω*_3_ have the form:


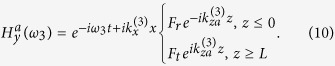


Here 
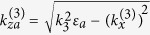
 is the longitudinal wave number of the wave at frequency *ω*_3_ in the homogeneous media. Enforcing the boundary conditions of field continuity at the interfaces of the stacked layers and using the modified TMM, we obtain the nonlinear scattering coefficients *F*_*r*_ at *z* < 0 and *F*_*t*_ at *z* > *L* describing the field of frequency *ω*_3_ emitted from the stack into the surrounding homogeneous medium.


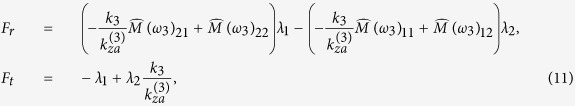


where


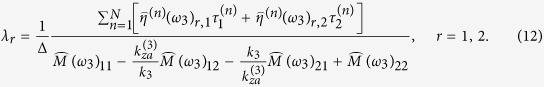


Let us note that *F*_*r,t*_ remain finite despite the coefficients 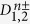 have poles at 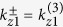. It was demonstrated that the coefficients 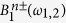 have the same singularity as 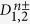 and their combined contribution is finite at all frequencies and incidence angles.

The analytical solutions for the coefficients *F*_*r,t*_ obtained in this section have allowed us to examine the mechanisms of nonlinear scattering in active semiconductor-dielectric stack.

## Simulation Results and Discussion

**Figure 3:**
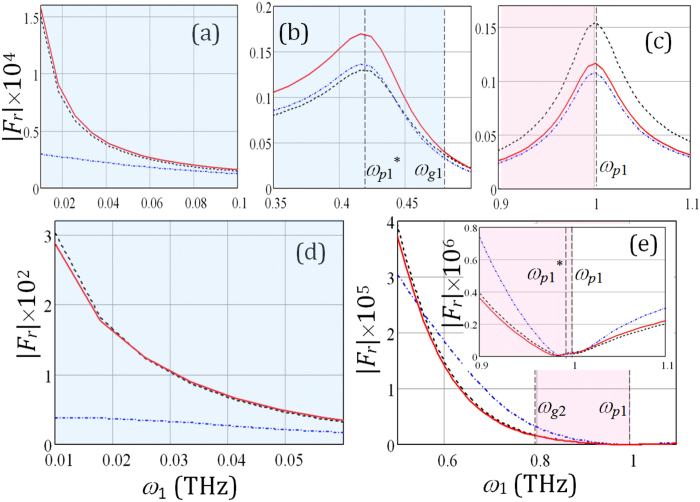
The field intensity |*F*_*r*_| of the waves, emitted from the passive and loss-compensated active stacks at frequency *ω*_3_ = *ω*_1_ + *ω*_2,_ versus a pump wave frequency *ω*_1_ at the other pump wave frequency (**a**–**c**) - *ω*_2_ = 0.573 THz and (**d**,**e**) - *ω*_2_ = 0.981 THz.

The simulated nonlinear scattering coefficients |*F*_*r*_| of the combinatorial frequency emitted from the passive (dash-dot blue curve) and active (solid red curve for two pump waves incident from the left, and dashed black curve for two pump waves incident from the right) stacks with the same parameters as above are displayed in Fig. 3 versus swept frequency *ω*_1_ of one pump wave incident at Θ_*i*1_ = 30°. The other pump wave, incident at Θ_*i*2_ = 60°, has two fixed frequencies *ω*_2_ such that |*R(ω*_2_)| has minima.

It can be seen that coefficients |*F*_*r*_| reach their peaks at the frequencies corresponding to the minima of |*R(ω*_1_)| while the peak magnitudes vary in accord with |*R(ω*_3_)|^13^. For the thin fine-stratified semiconductor-dielectric structure the maxima of the |*F*_*r*_| occur at *ω*_1_ = *ω*_*p*1_*, where *ω*_*p*1_* = *ω*_*p*1_−*ω*_*2*_ (Fig. 3(b)). Moreover, the field intensities |*F*_*r*_| have spikes at the semiconductor layer plasma frequency *ω*_*p*1_ as shown in Fig. 3(c) and inset of Fig. 3(e). This effect is attributed to the resonance field enhancement of the pump waves. The numerical results indicate that the introduction of gain dielectric material inside the stack can increase the efficiency of the CFG. The significantly higher intensity |*F*_*r*_| of combinatorial frequency emitted in the reverse direction of the *z*-axis was obtained at low frequencies (Fig. 3(a,d)) and in the proximity of resonant frequencies *ω*_*p*1_ and *ω*_*p*1_* (Fig. 3(b,c)). At the same time, when *ω*_2_ ≈ *ω*_*p*1_ the emission at *ω*_1_ > *ω*_*g*1_ is higher for the passive stack than that for the active one as evidenced by Fig. 3(e). This fact indicates that in the proximity of *ω*_*p*1_ the collision frequency has noticeable effect on longitudinal wavenumber of the uniaxial fine-stratified semiconductor medium as it was mentioned before. It was obtained that the dependencies |*F*_*t*_(*ω*)| have the same character, but the combinatorial frequency emission in the forward direction is slightly higher. In contrast to the passive stacks, the frequency mixing in the active systems exhibits the nonreciprocity especially in the proximity of the resonant frequencies at *ω*_1_ = *ω*_*p*1_ and *ω*_1_ = *ω*_*p*1_* as seen in Fig. 3(b,c). It is also noteworthy that for the wavelengths that are about 10 times larger than the total thickness of the structure the nonreciprocity of the emission in the forward direction (|*F*_*t*_|) is almost negligible.

**Figure 4:**
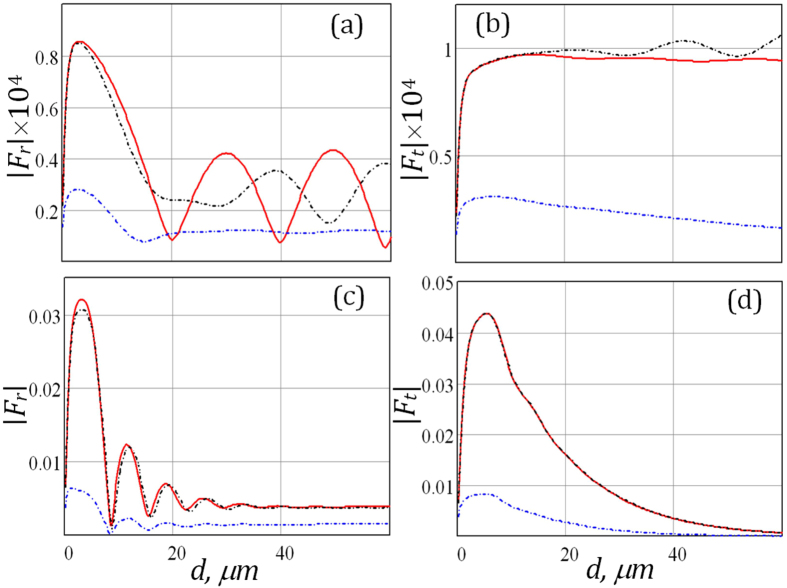
The field intensity of the waves emitted from the passive and loss-compensated active stacks at frequency *ω*_3_ = *ω*_1_ + *ω*_2_ in the reverse (**a**,**c**) and forward (**b**,**d**) directions of the *z*-axis. The structure was illuminated by pump waves with frequencies *ω*_1_ = 0.02 THz, (**a**,**b**) - *ω*_2_ = 0.573 THz (**c**,**d**) - *ω*_2_ = 0.981 THz; at Θ_i1_ = 30°, Θ_i2_ = 60°. The stack contains *N* = 40 unit cells, *α* = 3/7, *ν*_1_ = 0.05 THz.

The efficiency of nonlinear interactions in periodic layered structures significantly varies with the stack size^3^. This can be the result of the increased number of unit cells in the stack or variations in the thicknesses of the constituent layers. The |*F*_*r,t*_| simulated at the variable thickness of the unit cell are displayed in Fig. 4 for the passive and active stacks of the layers and two sets of pump wave frequencies. The nonlinear scattering coefficients have nonmonotonic dependences on the thickness *d*. However, it is necessary to remark that *ω*_3_ emission from the active stack exhibits noticeable growth almost for all unit cell thicknesses. The maximal efficiency of generated wave is observed for the active periodic stacks with *d* ≈ 3–6 *μm*. However, the dependences |*F*_*t*_(*ω*_1_)| for an active stack illuminated by the second pump wave with frequency *ω*_2_ = 0.573 THz (Fig. 4b) initially increase with the thickness *d* but reach saturation due to the decay of the pump waves tunnelling through the system. It can be seen that in this case the difference between the magnitudes of nonlinear response for passive and active stacks rises with the thickness of the unit cell. Figure 4(c) shows that magnitude of |*F*_*r*_| for *ω*_2_ = 0.981 THz gradually decreases and as in the case of passive structure the mean level of |*F*_*r*_| for an active stack becomes practically independent of thickness *d*. This implies that only a limited thickness of the fine-stratified stack influences the CFG, whereas contribution of the rest of the structure becomes insignificant due to the pump wave attenuation. The simulation results in Fig. 4(d) demonstrate that intensities |*F*_*t*_| steadily decrease at larger *d*. It is noteworthy that the weakest nonreciprocity of the nonlinear response corresponds to the second set of pump wave frequencies (*ω*_2_ ≈ *ω*_*p*1_). However, we have demonstrated that to obtain the stronger nonreciprocity we should decrease the number of the unit cells.

## Conclusions

In summary, we have explored the CFG by the non-Hermitian loss-compensated multilayer semiconductor-dielectric stack. We have demonstrated that incorporating gain within the multilayer system renders the compensation of semiconductor loss and significantly enhances the nonlinear interactions in the semiconductor stacks with resistive nature of nonlinearity. Depending on the pump wave frequencies and total stack size the noticeable nonreciprocity of the CFG can be achieved.

## Additional Information

**How to cite this article:** Shramkova, O. V. and Tsironis, G. P. Nonreciprocal nonlinear wave scattering by loss-compensated active hyperbolic structures. *Sci. Rep.* **7**, 42919; doi: 10.1038/srep42919 (2017).

**Publisher's note:** Springer Nature remains neutral with regard to jurisdictional claims in published maps and institutional affiliations.
